# Neoadjuvant chemotherapy combined with radical surgery for stage IB2/IIA2 cervical squamous cell carcinoma: a prospective, randomized controlled study of 35 patients

**DOI:** 10.1186/s12957-021-02318-y

**Published:** 2021-07-12

**Authors:** Huang Jing, Wu Xiuhong, Yu Ying, Liao Zhenrong, Cheng Xiyun, Luo Deping, Shen Changmei, Wang Qi, Peng Tao, Pan Yiyun

**Affiliations:** 1 Department of Gynecology and Oncology, Ganzhou Cancer Hospital, Ganzhou, 341000 China; 2 Radiotherapy Center, Ganzhou Cancer Hospital, Ganzhou, 341000 China; 3 Department of Chemotherapy Center, Ganzhou Cancer Hospital, No.19, Hua Yuan Qian Road, Ganzhou, 341000 Jiangxi Province China

**Keywords:** Adjuvant therapy, Cervical squamous cell carcinoma, Efficacy, Neoadjuvant chemotherapy, Radical surgery

## Abstract

**Objective:**

This study aimed to evaluate the clinical outcomes for patients with stage IB2/IIA2 cervical squamous cell carcinoma treated with neoadjuvant chemotherapy combined with radical surgery.

**Methods:**

A total of 68 patients with cervical squamous cell carcinoma were randomly divided into the experimental group (*n* = 35) and the control group (*n* = 33). The patients in the experimental group received paclitaxel plus cisplatin neoadjuvant chemotherapy for two cycles, then underwent radical hysterectomy and bilateral adnexectomy at 2 weeks post-chemotherapy. The control group only underwent radical hysterectomy and bilateral adnexectomy after the diagnosis of cervical squamous cell carcinoma. The toxic and side effects of chemotherapy in the experimental group were observed. Also, the operation method, operation time, blood loss, grade of wound healing, complications, and postoperative pathology were noted in the two groups. Primary foci and pelvic lymph node recurrence and distant metastasis were observed, and 3-year and 5-year survival rates were calculated.

**Results:**

Only one patient in the experiment had grade III bone marrow suppression; no other grade III and IV chemotherapy toxic reactions were observed. The operation was successfully completed in all patients. The operation time, intraoperative blood loss, placement of the ureteral catheter, bladder injury, ureteric injury, postoperative urinary tub, pelvic drainage tube indwelling time, anal exhaust time, postoperative complications, and metastatic ratio of lymph nodes were not significantly different between the two groups (*P* > 0.05). The number of dissected lymph nodes, deep myometrial invasion, and vascular tumor emboli showed a significant difference in the experimental group compared with the control group (*P* < 0.05). The 3-year disease-free survival (82.9% vs 81.9%), 5-year disease-free survival (71.4% vs 60.6%), 3-year overall survival (91.4% vs 87.8%), and 5-year overall survival (82.9% vs 75.6%) were not statistically significantly different between the experimental group and the control group (*P* > 0.05).

**Conclusions:**

Neoadjuvant chemotherapy in IB2/IIA2 stage cervical squamous cell carcinoma showed low toxic side effects. Radical surgery after chemotherapy is safe and feasible. It plays a coordinating role in reducing the tumor infiltration depth of the deep muscle layer and the incidence of vascular tumor emboli, reducing the use of postoperative adjuvant therapy, and improving the quality of life of patients, but does not improve the 3-year/5-year survival rate.

## Introduction

The prevalence of cervical squamous cell carcinoma and the number of young patients continue to increase annually [1, 2]. Locally advanced cervical squamous cell carcinoma is stage IB2/IIA2 cervical squamous cell carcinoma according to the International Federation of Gynaecologists and Obstetricians (FIGO 2009) [3]. Comprehensive treatment is the main treatment mode of locally advanced cervical squamous cell carcinoma or advanced cervical squamous cell carcinoma [4]. Many scholars believe that preoperative neoadjuvant chemotherapy (NACT) is desirable. It can improve the surgical resection rate of patients; decrease the incidence of parathymic infiltration, vascular thrombin level, and local and distant lymph node metastasis; and greatly reduce the incidence of postoperative pathological high-risk factors. It can also enable many young patients with early cervical squamous cell carcinoma to retain fertility, but its role in improving prognosis is still controversial [5–7]. Several meta-analyses evaluated the prognosis of neoadjuvant chemotherapy combined with surgical treatment and initial surgical treatment, and the results were different [8–10].

Patients do not receive any treatment before NACT; therefore, the vascular bed of the tumor is not destroyed, and chemotherapy drugs can easily enter the internal regions of tumors. The high blood concentration of the tumor can improve the response to chemotherapy and has a high tolerance to chemotherapy [11, 12]. Lesions sized > 4 cm, deep myometrial infiltration, and vascular cancer thrombus are intermediate-risk factors for the recurrence of locally advanced cervical squamous cell carcinoma. Lymph node metastasis and positive margin are high-risk factors for recurrence [13–15]. Whether NACT can reduce the incidence of moderate- and high-risk factors in postoperative pathology, improve the prognosis, and improve the survival rate in patients with locally advanced cervical squamous cell carcinoma is worthy of a prospective, randomized controlled clinical study.

## Materials and methods

### Ethics statement

This study received the Ganzhou Cancer Hospital Ethical Committee approval (Number: 2018A152), and the study was conducted following the principles of the Declaration of Helsinki regarding research involving human participants. Each patient provided written informed consent to participate after the nature of the study was explained to them.

### Selection criteria

#### Inclusion criteria

(1) All patients diagnosed with cervical squamous carcinoma based on the pathological examination and having stage IB2/IIA2 cervical squamous cell carcinoma according to the FIGO 2009 staging system; (2) Karnofsky performance score (KPS) ≥ 80; (3) initial diagnosis and first treatment; (4) the youngest being 18.0 years old and the oldest being 51.0 years old; (5) patients without other types of malignant tumors, and not including heart, liver, lung, and other important organ diseases; (6) patients whose expected survival time was more than 1 year; and (7) all patients voluntarily participating and signing informed consent.

#### Exclusion criteria

(1) Pathology-confirmed other types of cervical cancer, or not IB2/IIA2 squamous cell carcinomas; (2) no initial diagnosis and first treatment; and (3) patients with other types of tumors, including heart, liver, lung, and other important organ diseases, who could not tolerate chemotherapy and surgery.

### Clinical data

This was a prospective, clinical, randomized controlled trial. From January 2010 to September 2015, 68 patients with locally advanced cervical squamous cell carcinoma were enrolled in the study. They were randomly divided into two groups: the experimental group (*n* = 35) and the control group (*n* = 33). The patients in the experimental group received paclitaxel plus cisplatin neoadjuvant chemotherapy for two cycles, then underwent radical hysterectomy and bilateral adnexectomy combined with total pelvic lymph node dissection at 2 weeks post-chemotherapy. The control group only underwent radical hysterectomy and bilateral adnexectomy combined with total pelvic lymph node dissection after the diagnosis of cervical squamous cell carcinoma. All patients underwent computed tomography (CT) or magnetic resonance imaging (MRI) examination to determine tumor size and lymph node metastasis. No statistically significant difference was found in age, KPS score, pathological grade, tumor size, and clinical stage between the two groups (*P* > 0.05) (Table 1). All the 35 patients in the experimental group received two times of NACT before surgery.

**Table 1:** General comparison/patient (%)

Characteristic	Experimental group (***n*** = 35)	Control group (***n*** = 33)	***t***/***χ***^**2**^	*P*
Age (years)	45.32 ± 7.36	46.47 ± 8.12	0.61	0.54
KPS score				
≥90	23 (65.7)	20 (60.6)		
80–89	12 (34.3)	13 (39.4)	0.19	0.66
Grade				
High	2 (5.7)	2 (6.1)	0.16	1.00
Moderate	21 (60)	20 (60.6)		
Low	12 (34.3)	11 (33.3)		
Tumor size	4.81 ± 0.63	4.72 ± 0.74	0.54	0.59
Lymph node metastasis	10 (28.6)	9 (27.2)	0.01	0.91
FIGO stage				
IB2	16 (45.7)	15 (45.5)	0.001	0.98
IIA2	19 (54.3)	18 (54.5)		
HPV status				
High risk	12	15	0.288	0.59
Low risk	7	6		
Negative	16	12		

### Chemotherapy methods

Paclitaxel was combined with cisplatin in NACT. The specific regimen was as follows: paclitaxel 135–175 mg/m^2^ intravenous (i.v.) for 3 h and cisplatin 50–75 mg/m^2^ intravenous (i.v.). The heart rate, pulse, blood pressure, respiration, and other vital signs were closely observed during chemotherapy (3 weeks for a treatment course). A CT or MRI examination was performed after two courses to evaluate tumor regression.

### Surgical methods

After 2 weeks of rest following chemotherapy, the patients in the experimental and control groups underwent radical hysterectomy and bilateral adnexectomy combined with total pelvic lymph node dissection after the diagnosis of cervical squamous cell carcinoma. Para-aortic lymph node resection and sampling biopsy were performed when necessary. For patients less than 45 years old, one or both ovaries were preserved, and the ovarian suspension was performed simultaneously. For patients aged ≥ 45 years, bilateral adnexectomy was performed simultaneously, with frozen surgical margin and routine pathological examination of postoperative specimens.

### Principles and methods of adjuvant therapy

Patients with tumor diameter ≤ 4 cm, stromal invasion < 1/3, no vascular tumor emboli, and negative pelvic lymph nodes did not receive adjuvant therapy. Patients with tumor diameter > 4 cm, stromal invasion > 1/3, and vascular tumor emboli received postoperative adjuvant radiotherapy. Patients with positive pelvic lymph nodes received concurrent radiotherapy and chemotherapy. Three-dimensional intensity-modulated radiation therapy was used; the target area included pelvic lymphatic drainage area plus cervical tumor bed plus vaginal stump (DT 45–50.4 Gy/25–28F). Concurrent chemoradiotherapy was performed with cisplatin 30 mg/(m^2^ week) five times.

### Follow-up

The outcome measures included the toxic and side effects of chemotherapy, operation mode, operation time, bleeding volume, complications, and postoperative pathology. The recurrence and distant metastasis of primary and pelvic lymph nodes were observed. All patients were followed up every 3 months for the first 2 years after treatment, every 6 months for 2–5 years, and every year thereafter.

### Statistical analysis

All data were processed using IBM SPSS Statistics 22.0 statistical software. The data were expressed as $\overline{\mathrm{x}\ }$ ± s, and the comparison between the two groups was made using the *χ*^2^ test and *t* test. The survival rates and median survival times were estimated using the Kaplan–Meier method, and survival curves were generated. The log-rank test was used to analyze survival data.

## Results

### Toxic and side effects of chemotherapy in the experimental group

One patient had grade 3 myelosuppression. No grade III–IV gastrointestinal reactions, liver and kidney toxicity, and peripheral neurotoxicity were observed (Table 2). The evaluation of therapeutic effect in 35 patients after 2 courses of NACT revealed the following: CR 0 (0.0%), PR 28 (71.4%), NC 7 (28.6%), and PD 0 (0.0%).

**Table 2:** Toxic and side effects of chemotherapy in the experimental group/patient

Adverse reaction	I	II	III	IV
Myelo suppression	10 (28.6)	5 (14.3)	1 (2.9)	0 (0.0)
Gastrointestinal reactions	19 (54.3)	3 (8.6)	0 (0.0)	0 (0.0)
Liver and kidney toxicity	3 (8.5)	2 (5.7)	0 (0.0)	0 (0.0)
Peripheral neurotoxicity	2 (5.7)	1 (2.9)	0 (0.0)	0 (0.0)

### Analysis of operation situation

The experimental group included 20 cases of open operations and 15 cases of laparoscopic surgery; the control group included 21 cases of open operations and 12 cases of laparoscopic surgery, with no significant difference between the two groups (*χ*^2^ = 0.29, *P* = 0.59). The operation was successfully completed in all patients; no patients operated under video laparoscopy required conversion to open surgery. No iliac vascular injury was found in the two groups. The number of dissected lymph nodes during the operation was less in the experimental group than in the control group; the difference between the two groups was statistically significant (*P* < 0.05). Furthermore, no statistically significant differences in operation time, intraoperative blood loss, placement of the ureteral stent, bladder injury, ureteral injury, and so forth were observed (*P* > 0.05) (Table 3).

**Table 3:** Comparison of intraoperative conditions between the two groups/patient

Parameter	Experimental group (***n*** = 35)	Control group (***n*** = 33)	***t***/***χ***^**2**^	*P*
Operation time/min	188 ± 13	187 ± 32	0.17	0.87
Placement of ureteral stent	4 (11.4)	3 (9.1)	0.10	0.75
Number of dissected lymph nodes	22.51 ± 8.95	27.62 ± 8.83	2.37	0.02
Intraoperative blood loss	182.46 ± 191.37	175.13 ± 186.58	0.16	0.87
Bladder injury	1 (2.9)	1 (3.0)	0.002	0.97
Ureteral injury	2 (5.7)	1 (3.0)	0.29	0.59

### Analysis of operation quality

No statistically significant differences were found between the two groups in terms of the postoperative indwelling time of the urinary tube, pelvic drainage tube, anal exhaust time, and postoperative complication rate (*P* > 0.05) (Table 4).

**Table 4:** Comparison of intraoperative situation/patient (%)

Parameter	Experimental group (***n*** = 35)	Control group (***n*** = 33)	***t***/***χ***^**2**^	*P*
Catheter indwelling (days)	12.26 ± 3.64	11.58 ± 2.73	0.87	0.39
Drainage tube indwelling (days)	3.82 ± 1.90	3.57 ± 1.63	0.58	0.56
Anal exhaust (days)	2.68 ± 0.59	2.55 ± 0.68	0.84	0.40
Intestinal obstruction	2 (5.7)	1 (3.0)	0.29	0.59
Urinary fistula	2 (5.7)	2 (6.1)	0.004	0.95
Urinary retention	3 (8.6)	2 (6.1)	0.15	0.69
Urinary tract infection	7 (20)	6 (18.2)	0.04	0.85
Lymphatic cyst	5 (14.3)	6 (18.2)	0.19	0.67
Incision infection	2 (5.7)	1 (3.0)	0. 29	0.59

### Analysis of postoperative pathological factors

Both groups of patients were frozen during the operation to check the margins; the postoperative margins were negative. Statistically significant differences were observed in tumor size, deep muscular infiltration, and vascular cancer plug between the experimental group and the control group (*P* < 0.05). No significant difference was found in lymph node metastasis (*P* > 0.05) (Table 5).

**Table 5:** Comparison of postoperative pathology/patient (%)

Parameter	Experimental group (***n*** = 35)	Control group (***n*** = 33)	***t***/***χ***^**2**^	*P*
Tumor size	2.58 ± 0.32	4.72 ± 0.74	15.60	0.00
Lymph node metastasis	7 (20.0)	10 (30.3)	0.95	0.33
Deep muscle layer infiltration	19 (54.3)	26 (78.8)	4.49	0.03
Vascular tumor thrombus	2 (5.7)	8 (24.2)	4.58	0.03

### Postoperative adjuvant therapy

In the experimental group, 15 patients received no postoperative adjuvant therapy, while 20 patients received postoperative treatment with adjuvant radiotherapy (*n* = 13) and concurrent chemoradiotherapy (*n* = 7). In the control group, 5 patients received no postoperative adjuvant therapy, while 28 patients received postoperative treatment with adjuvant radiotherapy (*n* = 18) and concurrent chemoradiotherapy (*n* = 10). The difference in postoperative adjuvant therapy between the two groups was statistically significant (*P* < 0.05) (Table 6).

**Table 6:** Postoperative adjuvant therapy/patient (%)

Parameter	Experimental group (***n*** = 35)	Control group (***n*** = 33)	***χ***^**2**^	*P*
No therapy	15 (42.8)	5 (15.2)	6.19	0.01
Radiotherapy	13 (37.2)	18 (54.5)	2.04	0.15
Concurrent chemoradiotherapy	7 (20.0)	10 (30.3)	0.95	0.33

### Treatment effect

**Fig. 1:**
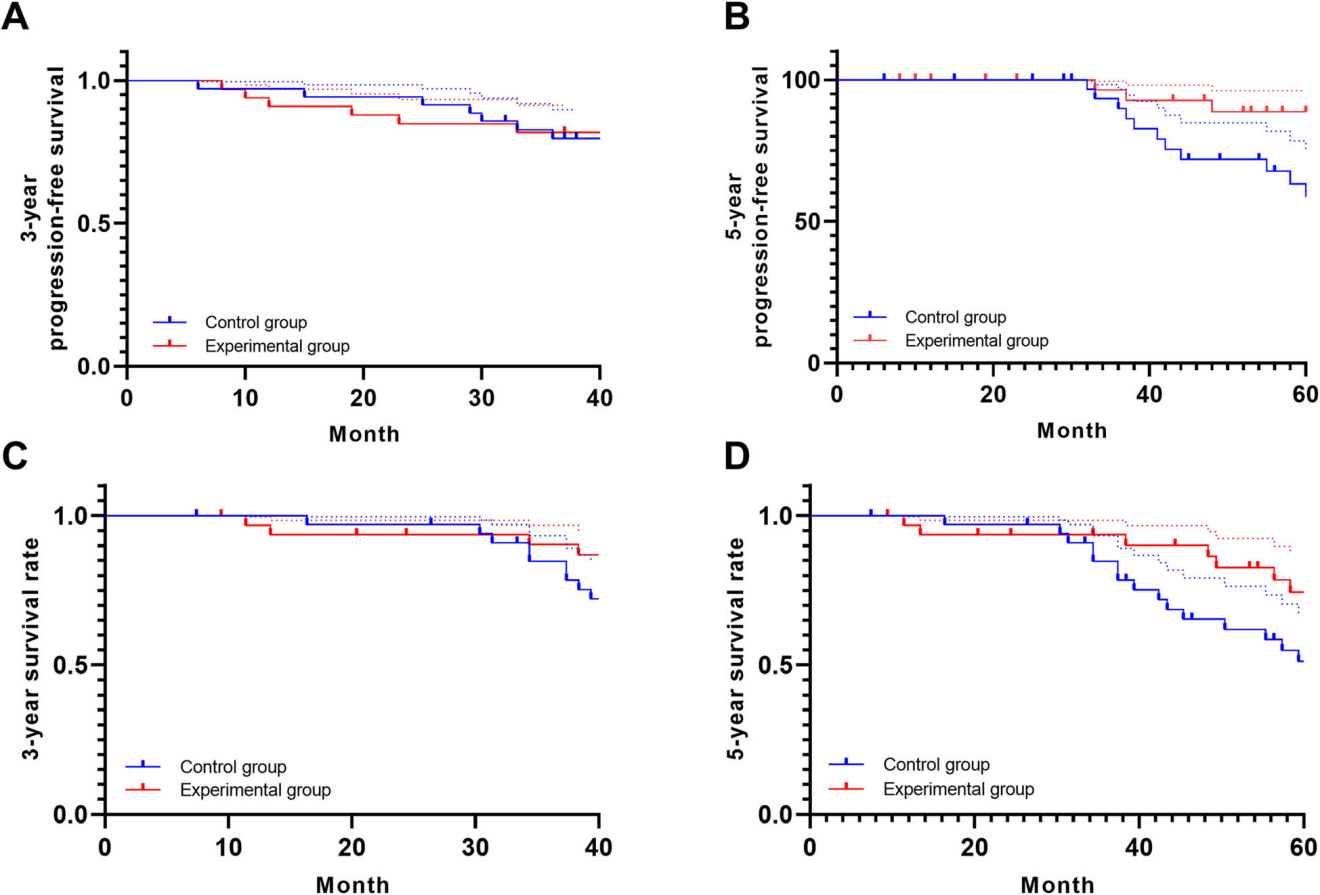
Different survival between the experimental group and the control group. **A** Three-year disease-free survival rate. **B** Five-year disease-free survival rate. **C** Three-year overall survival rate. **D** Five-year overall survival rate

All 68 patients were followed up from the end of treatment to September 2020; the follow-up rate was 100%. The median follow-up time was 58.4 months (25.0–103.0 months). The 3-year disease-free survival rate in the experimental group was 82.9% (29/35), while that in the control group was 81.9% (27/33), with no significant difference (*χ*^2^ = 0.016, *P* = 0.9002) (Fig. 1A). The 5-year disease-free survival rate was 71.4% (25/35) in the experimental group and 60.6% (20/33) in the control group, with no significant difference (*χ*^2^ = 0.100, *P* = 0.752) (Fig. 1B). The 3-year overall survival rate was 91.4% (32/35) in the experimental group and 87.8% (29/33) in the control group, with no significant difference (*χ*^2^ = 0.245, *P* = 0.620) (Fig. 1C). The 5-year overall survival rate was 82.9% (29/35) in the experimental group and 75.6% (25/33) in the control group, with no significant difference (*χ*^2^ = 0.256, *P* = 0.7089) (Fig. 1D).

### Comparison of treatment failure

A total of 23 patients in the 2 groups had recurrence or metastasis; the median time of recurrence or metastasis was 18.6 months (7.0–78.0 months). Among these, 16 patients had recurrence in the pelvic or vaginal stump, 3 patients had distant metastases (1 lumbar spine, 1 liver, and 1 mesenteric lymph node), and 4 patients had recurrence and distant metastases (2 lumbar spine, 1 liver, and 1 mesenteric lymph node) (Table 7). Further, 14 patients died (20.1%), and the median survival time was 24.3 months (11.6–52.7 months). All patients died due to tumor progression.

**Table 7:** Comparison of treatment failure/patient (%)

Parameter	Experimental group (***n*** = 10)	Control group (***n*** = 10)	***χ***^**2**^	*P*
Recurrence	15 (42.8)	5 (15.2)	6.19	0.01
Metastasis	13 (37.2)	18 (54.5)	2.04	0.15
Recurrence and metastasis	1 (10.0)	3 (20.1)	0.64	0.42

## Discussion

Squamous cell carcinoma is the most common pathological type of cervical cancer, followed by cervical adenocarcinoma and cervical adenosquamous carcinoma. Differences exist in the etiology, clinical features, response to radiotherapy and chemotherapy, and prognosis of patients with three pathological types of cervical cancer [16–18]. The National Comprehensive Cancer Network guidelines and FIGO guidelines do not distinguish the treatment of cervical adenocarcinoma and cervical adenosquamous carcinoma from that of cervical squamous cell carcinoma [3].

Many breakthroughs have been made in the diagnosis and treatment of cervical cancer in the last decade. Radical surgery and radiotherapy can increase the 5-year survival rate of early-stage cervical cancer to more than 90%, but the prognosis of locally advanced cervical cancer has not improved significantly. With surgery or radiation therapy alone, the 5-year survival rate is less than 50%; the potential high risk of recurrence and/or metastasis is still a risk factor that threatens the survival of such patients [19–21]. NACT combined with an extensive hysterectomy and pelvic lymph node dissection combined with para-aortic lymph node resection and sampling when necessary are continuously being explored to improve the survival rate of patients with locally advanced cervical cancer [22–24].

This trial simply examined the efficacy of NACT combined with radical surgery in treating locally advanced cervical squamous cell carcinoma. In theory, NACT can shrink tumors, improve parauterine infiltration, create conditions for surgery, and increase surgical resection rate; decrease tumor cell activity; and reduce the potential risk of intraoperative spread and postoperative recurrence and metastasis [7, 11–15]. Significant differences were found in the number of intraoperative dissected lymph nodes, deep muscle infiltration, and vascular tumor thrombus in the experimental group after NACT compared with the control group. Therefore, NACT could reduce the use of postoperative adjuvant therapy and improve the quality of life of patients.

After NACT in this trial, only one patient had grade III myelosuppression; no other grade III and IV chemotherapy toxic reactions were noted. No significant difference was found between the operation completion rate, operation time, intraoperative blood loss, ureteral stent placement, bladder injury, ureter injury, posterior urinary catheter, pelvic and abdominal drainage tube indwelling time, anal exhaust time, postoperative complication rate, and lymph node metastasis rate in the experimental group compared with the control group. The 3-year and 5-year disease-free survival rates and 3-year and 5-year overall survival rates in the experimental group were not significantly different from those in the control group. Therefore, NACT had few side effects in locally advanced cervical squamous cell carcinoma. Further, radical surgery after chemotherapy was safe and feasible, but it did not increase the 3-year or 5-year survival rate.

This was a prospective, clinical, randomized controlled trial. However, it had some shortcomings including small sample size, single-center design, and large time span; also, the results were somewhat biased. NACT, as a new auxiliary method in treating locally advanced cervical squamous cell carcinoma, is still in the stage of clinical trial research. Most of the existing studies are retrospective, with small sample size and short follow-up time. Moreover, the evaluations of tumor recurrence rate, tumor survival rate, and overall survival rate could not assess the overall advantages of NACT. Hence, more multi-center, large-sample, clinical, randomized controlled trials with longer follow-up time are needed [25–27].

The efficacy and prognosis of NACT in locally advanced cervical squamous cell carcinoma are related to many complex factors, and they are related to each other [28–30]. Therefore, an appropriate population size should be selected and the best individualized comprehensive treatment plan should be developed for each patient with locally advanced cervical squamous cell carcinoma to improve the quality of life and prognosis of the patients.

## Conclusion

Neoadjuvant chemotherapy in IB2/IIA2 stage cervical squamous cell carcinoma plays a coordinating role in reducing the tumor infiltration depth of the deep muscle layer and the incidence of vascular tumor emboli, reducing the use of postoperative adjuvant therapy. Neoadjuvant chemotherapy does not offer a significant survival benefit in IB2/IIA2 stage cervical carcinoma, but its low toxic side effects conduce to higher quality of patients’ life should deserve more attention of the clinician.

## Data Availability

Datasets are available on request from the corresponding author on reasonable request. The raw data and all related documents supporting the conclusions of this manuscript will be made available by the authors, without undue reservation, to any qualified researcher.
